# A second special issue of Standards In Genomic Sciences from the Genomic Standards Consortium

**DOI:** 10.4056/sigs.2295087

**Published:** 2011-11-22

**Authors:** Dawn Field, Renzo Kottmann, Norman Morrison, Peter Sterk

**Affiliations:** 1 NERC Centre for Ecology and Hydrology, Wallingford, UK; 2 Microbial Genomics Group, Max Planck Institute for Marine Microbiology and Jacobs University Bremen, Germany; 3 School of Computer Science, University of Manchester, Manchester, UK; 4 University of Oxford, Oxford e-Research Centre, Oxford, UK

This issue includes a series of papers from the second special issue of the Standards in Genomic Sciences (SIGS) journal organized by the Genomic Standards Consortium (GSC).

With this special issue, SIGS makes a significant advance towards its goal of providing a unique forum for publishing standards-compliant literature. With the publication of these articles SIGS is continuing to expand its scope and relevance to the wider scientific community working in the areas of genomes, metagenomes, metagenetics, 'omics and standard ways of describing, analyzing, distributing and publishing data.

While the content of SIGS to date has largely consisted of reports on genome sequencing projects - making SIGS the 3rd ranked journal for total number of genome publications to date - the longer-term goal of SIGS is to serve as an open-access, standards-supportive publication for all consensus-building communities working to develop standards and related infrastructure. Joint publication is a key step in the defining the shared interests and goals of communities. This issue contains a suite of multi-author, consensus-driven articles.

The papers in this special issue include a series of workshop reports, calls for adoption of new minimum information checklists, and a community call for open access to metagenomic samples. We look forward to seeing the international standards community continue to grow,  and we thank SIGS for providing a novel forum in which to publish critical advances in this field.

